# Behavioural Phenotypes and the Structure of Human Cognition

**DOI:** 10.1007/s11692-016-9399-y

**Published:** 2016-11-24

**Authors:** Dana Bentzen-Bilkvist, Andrea Migliano, Lucio Vinicius

**Affiliations:** Department of Anthropology, University College London, London, UK

**Keywords:** Cognition, Introspection, Imitation, Cooperation

## Abstract

Human cognitive uniqueness is often defined in terms of cognitive abilities such as introspection, imitation and cooperativeness. However, little is known about how those traits vary in populations or correlate across individuals. Here we test whether those three cognitive domains are correlated manifestations of an underlying factor, analogous to the psychometric ‘g’ factor, or independent ‘behavioural phenotypes’, analogous to the ‘Big-Five’ personality components. We selected eight variables measuring introspection and extraversion, verbal and physical imitation, cooperation and punishment, and evaluated their individual variability, domain-consistency and sub-structuring in a sample of 84 individuals. Results show high variation and limited clustering into three independent ‘behavioural phenotypes’ of introspection, imitation and cooperation. Only one significant correlation was identified (between two measures of extraversion), while other within-domain measures (introspection vs. extraversion, verbal vs. physical imitation, and cooperation vs. punishment) were not associated. Finally, no between-domain association was identified either through correlations or factor analysis. Overall, the results do not lend support to the hypothesis of a general ‘behavioural phenotype’ underlying individual behaviour. The independence of behaviours of introspection, imitation and cooperation may be the reason why individuals are able to adopt different behavioural strategies (combinations of behavioural phenotypes) and play distinct roles in the maintenance of human distinctive features such as hyper-cooperation and cumulative culture.

**Electronic supplementary material:**

The online version of this article (doi:10.1007/s11692-016-9399-y) contains supplementary material, which is available to authorized users.

## Introduction

Human societies exhibit unique features such as hyper-cooperation and cumulative culture (Burkart et al. 2014), often interpreted as the result of cognitive traits distinguishing humans from other species. Cumulative culture relies on high-fidelity transmission of socially shared knowledge (Dean et al. 2014), which is possible due to the human propensity to overimitate role models or copy actions irrelevant to achieving an explicit goal (Legare and Nielsen 2015). Introspection is another cognitive ability interpreted as distinctively human and our ‘default-mode processing’ allowing for mental displacement in space and time (Wilson et al. 2014), and even as the feature that allowed modern humans to outcompete Neanderthals (Mithen 1996). Extensive cooperation with unrelated and even unknown individuals and engagement in punishment of free-riders at an individual cost (Fehr and Gachter 2002) are two other features often claimed to differentiate humans from other species.

Although introspection, imitation, cooperativeness and other human cognitive abilities have been frequently investigated in isolation, little is known about how they are structured at individual level. For example, introspection, imitation and cooperation may be correlated in individuals, suggesting the existence of some general factor underlying human unique behavioural domains. In this case, we would be able to establish an analogy between those traits and the ‘general intelligence’ or ‘g’ factor often proposed to explain correlations across results of cognitive tasks (Deary 2001). Alternatively, traits such as introspection, imitation and cooperation may vary in mosaic fashion across individuals. This would suggest a parallel with psychological studies revealing a few independent personality components found in different combinations in individuals (Nettle 2006). Between the two extremes of full integration and mosaicism, a third possibility is partial linkage across some behavioural characteristics; for example, cooperative individuals may be more or less likely to overimitate, or more or less prone to engage in introspection. However, although many studies have examined the relationship between ‘agreeableness’, ‘openness’ and other ‘Big Five’ human personality components, there has been no analysis of associations between traits claimed to explain human cognitive uniqueness such as introspection, imitation or cooperation.

Peysakhovich et al. (2014) provided an initial attempt to investigate the structure and consistency of the human cooperative domain. Based on the application of multiple economic games and questionnaires to a large sample, they found that cooperative behaviours are consistent at individual level (co-operators or punishers in one economic game tend to cooperate or punish in other games and in real life) and over time (co-operators or punishers remained co-operators or punishers over time). They also showed that cooperative behaviours presented an internal subdivision: there was no association between measures of cooperation (offers in Dictator, Trust and Public Goods Games, behaviours and values of cooperation assessed via questionnaires) and measures of punishment (rejections in Ultimatum Game, behaviours and values of punishment from questionnaires). Based on this evidence, they proposed the existence of a human ‘cooperative phenotype’ characterised by consistency and sub-structuring of cooperative behaviours. A similar study based on 14 experiments has identified four independent subcomponents of prosociality across individuals (Bockler et al. 2016). Extrapolating from those results, we can ask whether other human cognitive domains, and even human cognition as a whole, display similar properties of individual variation, consistency and sub-structuring.

Here we test whether the cognitive domains of introspection and imitation can also be characterised as ‘behavioural phenotypes’. Those two domains were selected because they are frequently claimed to define human cognitive uniqueness, although they are by no means the only ones. We also assessed cooperative behaviours, in order to test for correlations across the three domains. The major aim of our analyses was to investigate whether the three behavioural domains are integrated in individuals, reflecting some general cognitive factor analogous to ‘g’, or vary independently in a similar way to the ‘Big Five’ personality components. We obtained multiple measures of imitation, introspection and cooperation and evaluated individual variability, domain-consistency and sub-structuring within each of our three selected behavioural phenotypes. Our results show that although all measures exhibit significant variability, there is some evidence (although preliminary and needing confirmation by further studies) that individual behaviour may be organised into independent ‘behavioural phenotypes’ of introspection, imitation and cooperation. However, behavioural integration does not seem to extend across the three domains into a ‘general’ behavioural phenotype analogous to ‘g’. The variability and independence of behaviours of introspection, imitation and cooperation may explain why individuals remain able to adopt different behavioural strategies (or combinations of behavioural phenotypes) and play different roles in the maintenance of human features such as hyper-cooperation and cumulative culture.

## Methods

We ran cognitive experiments and questionnaires (N = 84 subjects, 37 males; mean age = 27.8; age range 18–64) covering three behavioural domains: introspection, imitation and cooperation:

### Introspection

**Fig. 1:**
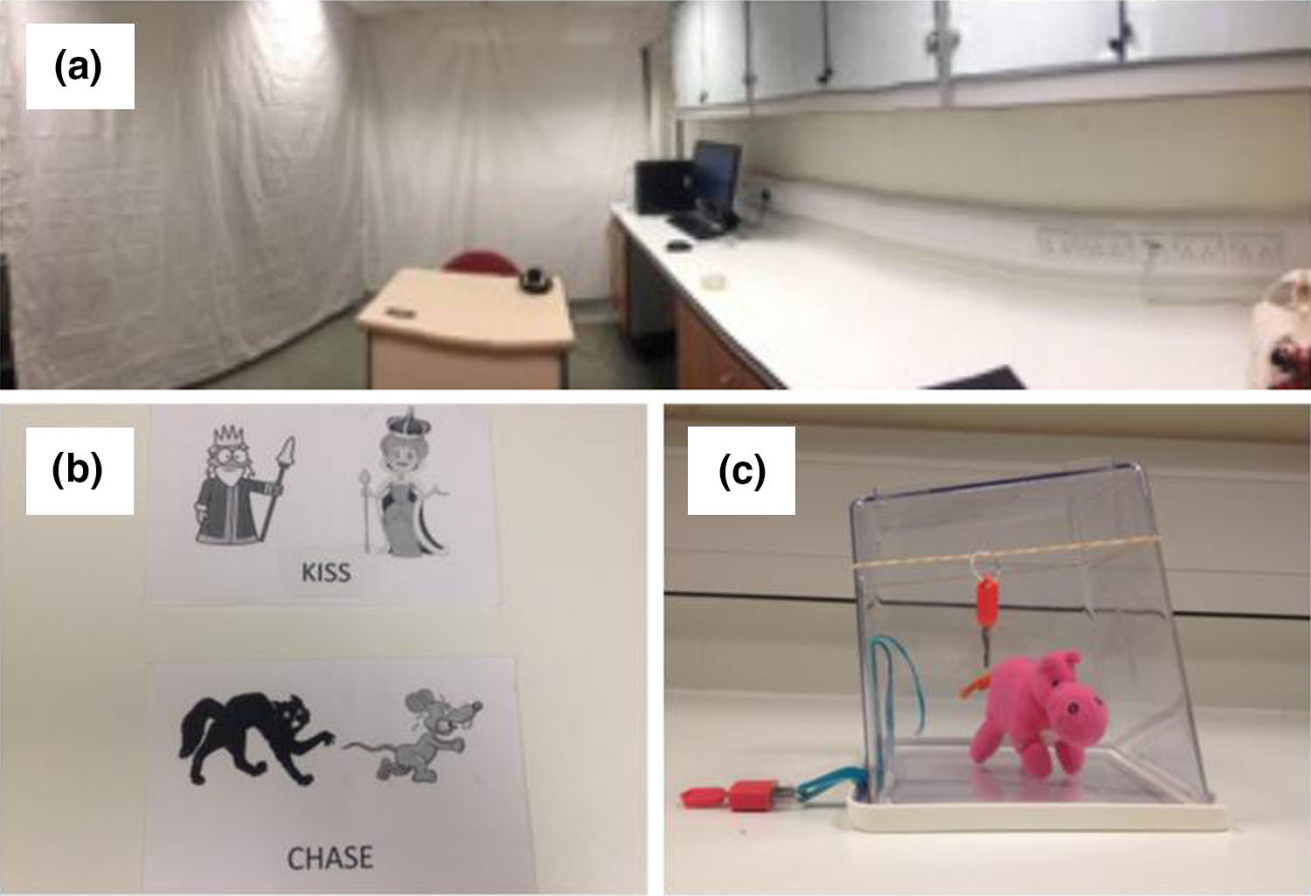
Experimental setting and apparatuses. **a** Laboratory setting for the Thinking Period experiment; **b** Syntactic alignment cards; **c** Imitation box (notice position of elastic band and padlock)

Thinking Period experiment (adapted from Wilson et al. 2014): participants were instructed to sit alone in a sterile lab (Fig. 1a) for 15 min and later were asked to rate two statements on a 5-point agreement scale (“Did you enjoy the 15-min thinking period?”; “Would you like to do another 15-min thinking period?”).

### Imitation

(1) Syntactic Alignment (adapted from Branigan et al. 2007): the experimenter selected a card showing two objects and a written verb, and then described the depicted event using one sentence, either in active or passive voice (example: ‘the ball broke the window’, or ‘the window was broken by the ball’; see examples in Fig. 1b). Then the participant was shown a new card and asked to do the same. The experimenter initiated ten descriptions alternating active and passive voice. The measure of syntactic alignment was the number of times the participant replicated the experimenter’s choice of active or passive voice. (2) Imitation Box (adapted from Horner and Whiten 2005): participants watched twice a short video of a person retrieving a toy from a transparent box after four actions, two necessary or ‘rational’ (flipping the box right side up, opening the latch) and two unnecessary or ‘irrational’ (removing an elastic band, unlocking a padlock attached to a ribbon; Fig. 1c). Participants were then presented with the same box and asked to retrieve the toy, and the number of copied unnecessary actions was counted. Finally we asked participants about how many actions they remembered from the video demonstration, and which ones they considered to be necessary to open the box. Interestingly, 50 participants believed that removing the padlock was necessary, and 9 thought that the removing the elastic was necessary. Analysing separately the subsample of individuals who were aware of the irrationality of both padlock and elastic removal (N = 31) did not change the overall results. For this reason, we only present results from the whole sample (N = 84).

### Cooperation

Participants played two classic economic games for a fee. (1) Dictator Game: Player A was given £5 and could share from 1p to £5 with an unknown Player B. (2) Ultimatum Game: Player A was given £5 to split with an anonymous Player B, who could either accept the offer (splitting the money as proposed by Player A) or reject it (in which case neither Player A or B received money). Sample size is n = 84 in the Dictator Game, but was split into two in the Ultimatum Game (42 subjects were selected as Player A, and 42 as Player B).

### Questionnaires

After the experiments, subjects were asked to rate statements on a 5-point agreement scale. The statements covered three topics: extraversion (Myers 1962), social media use (Wilson et al. 2014), and values and behaviours of cooperation and punishment (Peysakhovich et al. 2014).

### Variable Definition

From the experiments and questionnaires above, we derived eight variables assessing individual behaviour. Introspection domain: (1) Introspection Score (IS), sum of agreement ranks from two questions on the Thinking Period experiment; (2) Extraversion Score (ES), sum of ranks from nine questions; (3) Social Media Score (SM), sum of scores of eight questions. Imitation domain: (4) Syntactic Alignment Score (SA), number of times subjects copied the experimenter’s choice of active or passive voice; (5) Imitation Box Score (IB), number of irrational moves copied by participant. Cooperation domain: (6) Dictator Game offer (DG); (7) Ultimatum Game offer (UG); and (8) Behaviours of Punishment score (BP), sum of answers to three questions.

### Analyses

We ran non-parametric Kendall correlations between all eight variables, with Holm-Bonferroni correction for multiple testing (28 correlation tests). We also performed PCA and factor analysis to investigate possible underlying factors across domains.

## Results

### Behaviours Across Cognitive Domains are Variable and Typically not Extreme

**Fig. 2:**
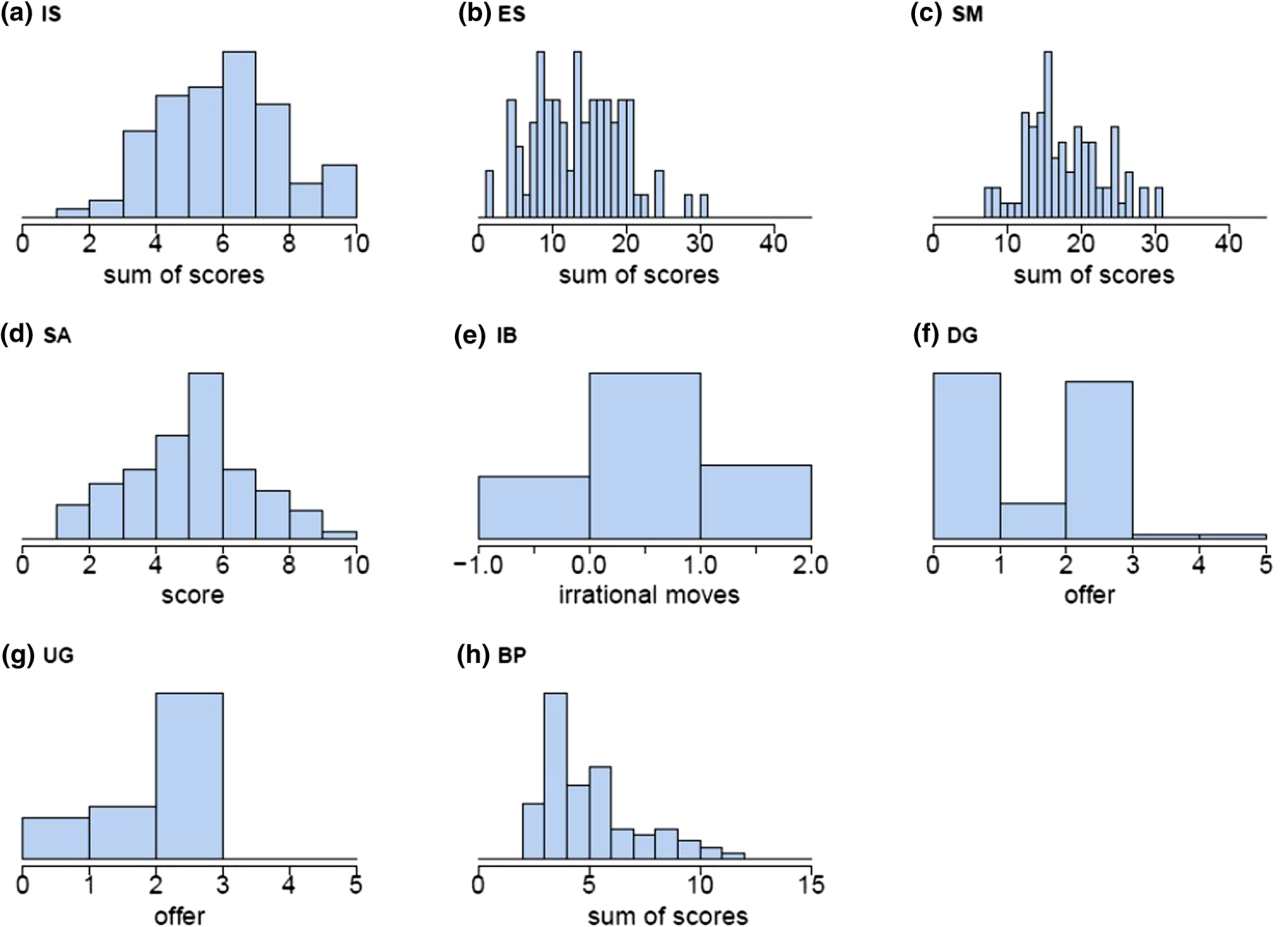
Distribution of introspection, imitation and cooperation variables. Histograms display distributions of nine variables: **a** Introspection score (IS), **b** Extraversion score (ES), **c** Social media score (SM), **d** Syntactic alignment score, **e** Imitation box score (IB), **f** Dictator game offer (DG), **g** Ultimatum game offer (UG), **h** Behaviours of punishment score (BP), **i** Values of punishment score (VP). Sample size is 101 individuals, except for UG where sample size is 50. For definitions of each variable see “Methods” section

All traits showed significant variation between extremes of introspection and extrospection, introversion and extraversion, overimitation and innovation, cooperation and selfishness, punishment and non-punishment (Fig. 2). There is significant variability in enjoyment of introspection, rejecting a previous suggestion (Wilson et al. 2014) that people generally dislike thinking periods (see Fox et al. 2014). Both measures of imitation showed intermediate peaks, indicating that extremes of overimitation or innovation are not the rule in humans. With the exception of Dictator Game offers (with two peaks near the minimum and egalitarian offers) and behaviours of punishment (with peaks at the lowest values), distributions are also approximately bell-shaped rather than characterised by extreme values. However, distributions of all variables (except Extraversion Score and Social Media Score) significantly deviate from normality (Shapiro–Wilk tests, *P* < 0.05).

### Intra-Domain Correlations Suggest Independent ‘Behavioural Phenotypes’

Despite extensive variation in all traits, only one significant correlation was identified between variables. The two measures of extraversion, Extraversion Score and Social Media Score, correlated significantly (Kendall tau = 0.24, z = 3.1, *P* = 0.04 after Holm–Bonferroni correction), but neither correlated with the Introspection Score. There was no correlation between verbal and physical imitation (Syntactic Alignment Score and Imitation Box). There was also a correlation between two measures of cooperation in economic games, Dictator Game offer and Ultimatum Game offer (Kendall tau = 0.36, z = 2.9, *P* = 0.0037), but it becomes non-significant after correction for multiple testing (*P* = 0.1). None of the remaining correlations achieved significance even before correction for multiple testing. In summary, correlation tests provide very limited evidence for internal structuring within the three domains of introspection, cooperation or imitation.

### No Evidence of a General ‘Behavioural Phenotype’

Next we tested for inter-domain correlations among introspection, imitation and cooperation variables, but none was significant (all *P* > 0.1 before Holm-Bonferroni correction). Lastly we searched for general factors underlying variation in individual behaviours. An exploratory PCA produced a first PC explaining only 23.8% of total variance (vs. 12.5% expected by random chance), and no clear drop in subsequent PCs (PC 2: 18.2%, PC 3: 16.2%). We also ran factor analyses with up to four factors (the maximum possible for eight variables), but none was significant (significance was at least *P* = 0.18), indicating no general association of variables across domains. PCAs and factor analyses remain not significant, even after removal of the two non-bell-shaped variables (Dictator Game offer and behaviours of punishment).

## Discussion

Our main finding is that there is no evidence that human behaviours across the domains of introspection, imitation and cooperation are structured by a pervasive ‘behavioural phenotype’ or underlying factor analogous to the ‘g’ factor. The lack of correlation between cooperation and punishment variables confirmed previous suggestions of a sub-structured ‘cooperative phenotype’ (Peysakhovich et al. 2014). The loss of significance of the correlation between Dictator Game and Ultimatum Game offers after correction for multiple testing may reflect a further differentiation between respectively ‘altruistic’ and ‘strategic’ prosocial behaviours (Bockler et al. 2016) or simply too much noise in our sample. Further testing with larger samples is needed to provide an answer to this question. The only significant correlation (between Extraversion Score and Social Media Score) was internal to the introspection domain. However, neither of those two measures of introversion showed an association with our measure of introspection (Introspection Score). This suggests that extraversion (the tendency to seek social interactions) and introspection (inward-directed thought) may coexist in the same individual. The lack of correlation between introspective and extraverted behaviours seems to be analogous to the independence of cooperation and punishment within the ‘cooperative phenotype’, thus providing partial evidence for internal sub-structuring of an ‘introspection phenotype’. Also, for the first time we have shown that that individual biases in physical (imitation box) and verbal (syntactic alignment) imitation seem to vary independently, suggesting that they may represent two separate aspects of a possible ‘imitation phenotype’. Finally, the lack of general integration across the three domains also implies that distinct combinations of innovative, imitative and cooperative behaviours are possible at individual level.

However, the results above must be interpreted with care. The lack of correlations and significant underlying factors are negative results. As such, although they are relevant for not supporting the hypothesis of a general ‘behavioural phenotype’, they cannot definitely reject its existence. More convincing demonstration would require larger sample sizes and further testing. Therefore, the main contribution of our study is to suggest that an underlying behavioural factor across the domains of introspection, imitation and cooperation cannot be as easily identified as the ‘g’ factor or ‘general intelligence’ factor, often revealed by psychometric studies based on sample sizes similar to ours.

The absence of convincing evidence for a ‘behavioural phenotype’ underling individual behaviour is nonetheless relevant to current debates over human cognitive uniqueness. Some studies have proposed that cognitive abilities such as overimitation, introspection and cooperativeness can distinguish humans from other species. However, chimpanzees, our closest relatives, also initiate and maintain cooperation (Suchak et al. 2014), exhibit cultural variation in the wild (Hobaiter et al. 2014), imitate group traditions (Whiten et al. 2009) and may act based on a theory of mind (Hare et al. 2001). In addition, there is significant overlap in cognitive tests between the two species. Experiments by Hermann et al. (2007) revealed individual success rates in children between 35 and 100% in social learning tests, 20–100% in communication tests, and 20–100% in theory of mind tests. In adult chimpanzees, figures were respectively 0–70, 20–90 and 20–75%. Consistent with those results, our tests revealed significant behavioural variability in the three domains, and a prevalence of intermediate values across measures. The results seem to rule out the possibility that human behaviour is generally characterised by a tendency towards extremes of introspection, imitation or cooperation. Together, the results point against the possibility of differentiating human from chimpanzee cognition simply in terms of the presence or absence of the three cognitive abilities.

We suggest that human cognitive uniqueness may be more easily defined by frequencies of behaviours at population level. In our sample, approximately 40% of individuals made egalitarian offers in the Dictator Game, 50% enjoyed introspection in the Thinking Period assay, and 77% copied at least one irrational move in the Imitation Box test. Previous studies claimed that cumulative culture and hyper-cooperation may depend on population size (Powell et al. 2009), and we extend this argument by proposing that they might also require a minimum number of co-operators and over-imitating individuals respectively. For example, agent-based simulations showed that about 80% of individuals should be co-operators (against 20% free-riders) in order for demand sharing, a possibly ancestral form of human hyper-cooperation, to be maintained in hunter-gatherer populations (Lewis et al. 2014). Similar arguments may hold for cumulative culture, with some studies proposing that it might depend on a combination of imitative behaviours (that provide fidelity to cultural transmission) and innovative behaviours (that allow cultural change to occur) (Legare and Nielsen 2015).

This proposal is compatible with individuals remaining able to adopt distinct decision-making strategies and social behaviours, and with independence and combinatorial variety of behavioural phenotypes. The apparent dissociation among introspection, imitation and cooperation behaviours in our sample contrasts not only with the psychometric ‘g’ factor, but also with a proposed inter-specific or ‘primate g’ or ‘g_s_’ (Reader et al. 2011), a general intelligence factor across primates derived from strong correlations among cognitive abilities such as social learning, innovation rates and tool use (the ‘cultural’ core of g_s_). The contrast between a general primate ‘g’ factor and our independent behavioural phenotypes may be explained by the different traits used to define human cognitive uniqueness, by differences between intra- versus inter-specific comparisons, or as discussed above, by noise in our particular sample.

Our study can be seen as a contribution of cognitive science to the general debate on the roles of modularity and integration in human evolution. Previous analyses have contrasted mosaicism versus developmental and functional integration in relation to skull morphology (Bastir 2008); grade shifts and mosaic evolution (Barton and Harvey 2000; Smaers et al. 2010) versus general scaling principles and developmental integration (Mota and Herculano-Houzel 2015) in the context of primate and human brain evolution; modular (Fodor 1983) versus connectionist models (Elman 2005) of brain function, among others. At the cognitive level, theories based either on massive modularity (Tooby and Cosmides 1992) or increased integration (Mithen 1996) have also been proposed but are difficult to experimentally verify. Explaining human cognitive uniqueness from an experimental perspective is a complex topic. Getting closer to an answer requires a better understanding of how components of human cognition, however defined, vary and relate at individual and population level. Our study design presents some limitations, including the lack of test–retest reliability measures (due to time constraints). Psychometric experiments can also suffer from the presence of floor or ceiling effects, which could be especially true for our Imitation Box experiment that included only three possible results. In the future, this could be solved through the application of multiple imitation tasks to each participant and calculation of a total individual imitation score. However, our variable distributions were never concentrated at lower or higher values and mode/median values were as a rule found in the mid-range, with the exception of the well-studied bimodal distribution of Dictator Game offer with peak values at 0 (selfish offer) and middle of the range (egalitarian offer). As discussed above, our negative results seem do not lend to support to the existence of a general ‘behavioural phenotype’, but do not allow definitive rejection of this hypothesis. Our study is therefore a starting point to psychometric investigations of traits claimed to define human cognitive uniqueness. Further studies of verbal, physical and other modalities of imitative behaviour, as well as other measures of introspection and introversion, are necessary to confirm whether ‘behavioural phenotypes’ other than cooperation characterise human cognition.

## Electronic supplementary material

Below is the link to the electronic supplementary material.

Supplementary material 1 (CSV 2 kb)
